# Maxillary Sinus Mucopyocele in a Fifty-eight-year-old man: A Possible Late Complication of Irradiation to Head and Neck

**DOI:** 10.5812/ircmj.17133

**Published:** 2014-07-05

**Authors:** Hadi Sharouny, Prepageran Narayanan

**Affiliations:** 1 Department of Otorhinolaryngology Head and Neck Surgery, Faculty of Medicine, Shiraz University of Medical Sciences, Shiraz, IR Iran; 2 Department of Otorhinolaryngology, Head and Neck Surgery, Faculty of Medicine, University of Malaya, Kuala Lumpur, Malaysia

**Keywords:** Mucocele, Maxillary Sinus, Paranasal Sinuses, Radiotherapy, Complications

## Abstract

**Introduction:**

A mucocele is an epithelial-lined, mucus-containing sac that can fill the sinus completely and expand gradually. Mucopyocele is an infected mucocele.

**Case Report:**

We presented a case of left maxillary mucopyocele in a 58-year-old man that developed after radiotherapy for nasopharyngeal carcinoma. Computed tomography scan showed opacification of the left maxillary sinus expanding through the medial wall of the antrum with thinning and destruction of the adjacent structures. Endoscopic marsupialization of the lesion and left partial maxillectomy were performed. The cystic mass had yellowish thick mucopurulent fluid that was completely drained.

**Conclusions:**

A few cases of sphenoid sinus mucocele as a late complication of radiation therapy have been reported. Maxillary mucocele and mucopyocele can be considered as one of the late complications of radiotherapy to head and neck as a result of occlusion of sinus ostia by scarred mucosa.

## 1. Introduction

Rollet introduced the term mucocele in 1896 and Oondi provided first histological description in 1901 (1). A mucocele is an epithelial-lined, mucus-containing sac that can fill the sinus completely and expand gradually (2). When the mucocele content becomes infected, the lesion is termed mucopyocele (3). The fronto-ethmoidal region is the most commonly affected anatomical area with the highest prevalence in ethmoid, sphenoid, and maxillary sinuses, consecutively (4). In a study by Lund, two-thirds of mucoceles were secondary to obvious predisposing factors such as infection, polyps, and trauma and one-third were idiopathic (5). In another study by Arrue et al. 50% of patients with mucocele had a history of prior infection, 25% had a history of trauma, and 10% had a prior allergic disorder (6). This case report aimed to draw attention to the maxillary sinus mucocele as a possible late complication of irradiation to head and neck.

## 2. Case Report

**Figure 1.**
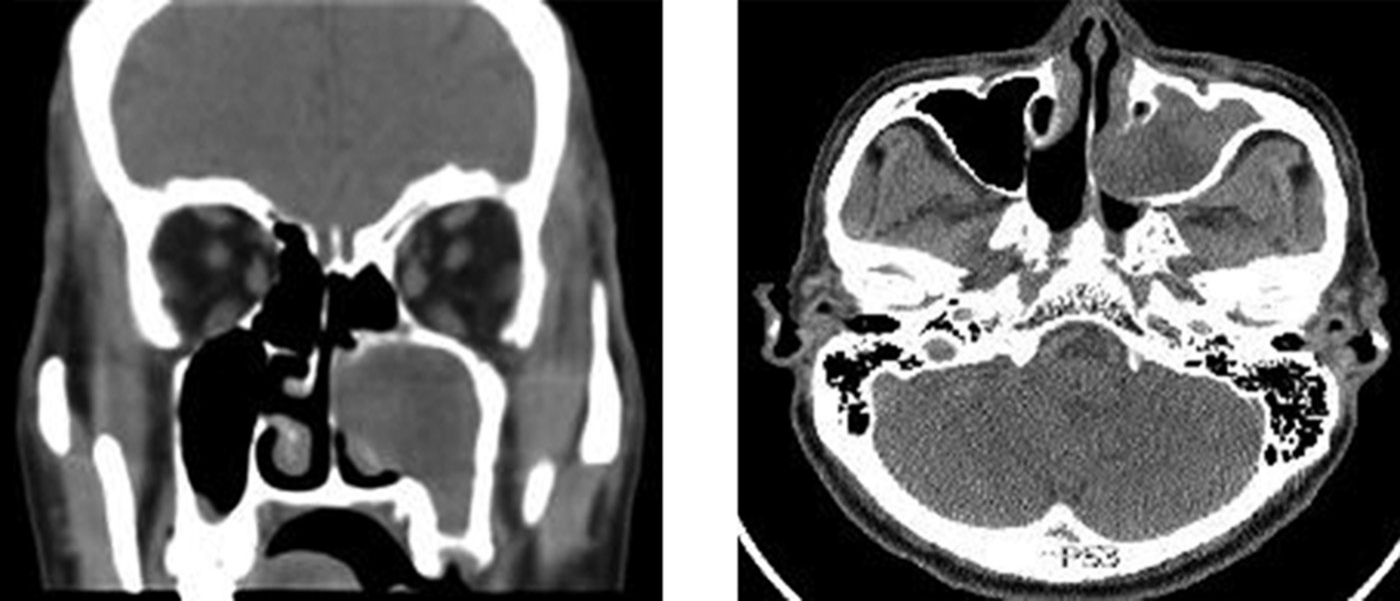
Computed Tomography Shows an Expansible Homogenous Opacity in the Left Maxillary Sinus Left: coronal plane; Right: axial plane.

A 58-year-old man with complaint of left nasal obstruction for eight years attended the otorhinolaryngology clinic of University Malaya Medical Centre in January 2013. There was no history of runny nose, nasal bleeding, sneezing, or loss of smell. He had received radiotherapy for nasopharyngeal carcinoma (NPC) twice, about 13 and three years ago. Patient was on fluticasone nasal spray without any improvement. On examination, there was no facial swelling. Anterior rhinoscopy showed deviation of the nasal septum to the right. Nasal endoscopy revealed a soft nontender mass without bleeding on probing that had occupied left middle meatus and had pushed the nasal septum to the right side. There was no mass at Rosenmuller’s fossa. Computed tomography (CT) images revealed a homogenous opacity in the left maxillary sinus with the mass that had extended medially; the mas had deviated the nasal septum to the right with thinning and destruction of the adjacent structures (Figure 1). Under general anesthesia, surgical decompression of the mass through an endoscopic approach was done. Thick yellowish mucopurulent fluid was aspirated from the mass. Endoscopic marsupialization was completed with microdebrider and left partial medial maxillectomy was performed. Patient had an uneventful postoperative course and was discharged from hospital on the following day. The patient had regular follow-ups with no evidence of recurrence.

## 3. Discussion

The late complications following radiotherapy for NPC are documented well (7). Scarring and obstruction of sinuses due to irradiation cause rhinosinusitis, which usually remains undetected and leads to the under-reporting of this complication (7). By obtaining serial CT images before and after irradiation of patients with NPC, Poter revealed a significant increase in mucosal abnormalities in the paranasal sinuses by irradiation (7). A study in Taiwan that used CT images to assess paranasal sinuses after radiotherapy for NPC revealed that chronic sinus disease was a prevalent late complication of radiotherapy for NPC and might persists for years before diagnosis (8). Searching PubMed database using the keywords including mucocele, sinus, and radiotherapy showed that mucocele developing after radiotherapy for NPC had been reported rarely (8-13). In a study in Singapore on ten cases of sphenoid sinus mucocele, three patients (30%) had a history of radiotherapy for treating NPC. Irradiation to the head and neck appeared to be a predisposing factor to sphenoid sinus mucocele (13). To the best of our knowledge, there are no prior reports of maxillary mucocele after radiotherapy and hence, this case seems to be the first case report of maxillary mucopyocele in a patient after irradiation to the head and neck. In literature review, only sphenoid sinus mucoceles were reported as the late complication of radiotherapy. All of these patients had NPC and had received radiotherapy (8-13). There is no report regarding frontal or ethmoidal mucoceles as the late complication of radiotherapy to head and neck.

Scarring of the mucosa after irradiation may cause occlusion of the sinus ostium, which leads to formation of mucocele (10). Sphenoidal sinus mucoceles developing after radiotherapy for NPC have been reported rarely. Maxillary sinus mucocele is also a possible late complication of radiotherapy to the head and neck. Paranasal CT is a useful tool in making the diagnosis. Biopsy is also required to diagnose recurrence or associated irradiation-induced tumors. Endoscopic marsupialization of mucocele is the best management and will confirm the diagnosis intraoperatively. Patients should be examined thoroughly in follow-ups to detect any signs of recurrence as early as possible. We suggest a multicentric study on a large number of mucoceles to show etiological link between radiotherapy and mucocele of different paranasal sinuses.
